# Early Learning Curve in the Assessment of Deep Pelvic Endometriosis for Ultrasound and Magnetic Resonance Imaging

**DOI:** 10.1155/2020/8757281

**Published:** 2020-09-26

**Authors:** T. Indrielle-Kelly, D. Fischerova, P. Hanuš, F. Frühauf, M. Fanta, P. Dundr, D. Lavu, D. Cibula, A. Burgetova

**Affiliations:** ^1^First Faculty of Medicine, Charles University in Prague, Czech Republic; ^2^Department of Obstetrics and Gynecology, Burton Hospitals NHS, Belvedere Road, Burton-on-Trent, DE13 0RB West Midlands, UK; ^3^Department of Obstetrics and Gynecology, First Faculty of Medicine, Charles University and General University Hospital in Prague, Apolinářská 18, 128 08, Czech Republic; ^4^Department of Radiology, First Faculty of Medicine Charles University and General Faculty Hospital in Prague, U Nemocnice 2, 128 00 Prague, Czech Republic; ^5^Department of Pathology, First Faculty of Medicine, Charles University and General University Hospital in Prague, U Nemocnice 499, 128 08 Prague, Czech Republic; ^6^ACALM Study Unit, Birmingham, UK

## Abstract

**Purpose:**

We aimed to compare the learning curves of an ultrasound trainee (obstetrics and gynecology resident) and a radiology trainee when assessing pelvic endometriosis.

**Methods:**

Consecutive patients with suspected endometriosis were prospectively enrolled in a tertiary center. They underwent an ultrasound and magnetic resonance imaging preoperatively, which was reported according to the International Deep Endometriosis Analysis (IDEA) group consensus. Trainees reported on deep endometriosis (DE), endometriomas, frozen pelvis, and adenomyosis. Using the Kappa agreement, their findings were compared against laparoscopy/histology and expert findings. The learning curve was considered positive when performance improved over time and indeterminate in all other cases.

**Results:**

Reports from thirty-five women were divided chronologically into 3 equal blocks to assess the learning curve. For ultrasound, trainee versus expert showed a positive learning curve in overall pelvic DE assessment. There was an excellent agreement for adenomyosis (Kappa = 1.00, *p* = 0.09), frozen pelvis (Kappa = 0.90, *p* = 0.01), bowel (Kappa = 1.00, *p* = 0.01), and bladder DE assessment (Kappa = 1.00, *p* = 0.01). Endometrioma and uterosacral ligament assessment showed an indeterminate curve. For radiology, trainee versus expert showed a positive curve when detecting adenomyosis (Kappa = 0.42, *p* = 0.09) and bladder DE (Kappa = 1.00, *p* = 0.01). The assessment of endometriomas, frozen pelvis, overall pelvic DE, bowel, and uterosacral ligament DE showed indeterminate curve. Agreement between trainees and laparoscopy/histology showed a positive curve for bladder (both) and frozen pelvis (ultrasound only).

**Conclusion:**

A positive learning curve can be seen in some areas of pelvic endometriosis mapping after as little as 35 cases, but a bigger caseload is required to demonstrate the curve in full. The ultrasound trainee had positive learning curves in more anatomical locations (bladder, adenomyosis, overall bowel DE, frozen pelvis) than the radiology trainee (bladder, adenomyosis), which could be down to individual factors, differences in training, or the imaging method itself.

## 1. Introduction

Accurate preoperative mapping of pelvic endometriosis is crucial for individualized treatment. It is important that professionals reading images report systematically on the presence of adenomyosis, endometriomas, frozen pelvis (as an indirect sign of endometriosis [1]), and deep endometriosis (DE) lesions. Ultrasound and magnetic resonance imaging (MRI) are predominantly used for the evaluation of the pelvic endometriosis, but only for ultrasound, there is an internationally accepted consensus on terms, definitions, and measurements, the International Deep Endometriosis Analysis (IDEA) [2] group consensus. There is no similar document which guides MRI reporting; however, the European Society of Urogenital Radiology (ESUR) [3] published guidelines on the technical protocol for pelvic MRI in endometriosis.

Ultrasound is widely accepted as a method of choice for detecting endometriomas [4], and it was shown to have similar accuracy to MRI in diagnosing adenomyosis [5] and DE [6]. Despite good evidence on the accuracy of ultrasound [6], its wide availability, and no contraindication for use, it is frequently not the diagnostic modality of choice due to various reasons. One such reason is the lack of training and skills in this area. In order to even consider a new imaging method, one has to contemplate the necessary training requirement, characterised by the learning curve.

The learning curve can be described as an improvement in the performance of a given task. In ultrasound, this would consist of not only gaining theoretical knowledge and its application in pattern recognition but also learning probe manipulation, which requires good hand-eye coordination and manual dexterity. For MRI, the learning curve may be shorter since manual dexterity is not necessary. Accuracy is expected to plateau after a certain number of cases.

In this paper, we aimed to compare the learning curve of an obstetrics and gynecology trainee (O&G) using ultrasound and a radiology trainee using MRI when evaluating pelvic endometriosis, where expert reports and histologically confirmed laparoscopic findings served as reference standards.

## 2. Methods

This prospective study was conducted at a tertiary referral endometriosis center. It is aimed at comparing the learning curve of an ultrasound and a radiology trainee when assessing pelvic endometriosis (adenomyosis, endometriomas, frozen pelvis, and DE) in the same cohort of patients using one predefined protocol, which was based on the International Deep Endometriosis Analysis (IDEA) group consensus [2] adapted for MRI, as per Indrielle-Kelly et al. [7]. Diagnostic performances of trainees were compared against the accuracy of in-house ultrasound and radiology experts and also against histologically confirmed laparoscopic findings.

There are several ways of assessing a learning curve, and in this study, we used the following model which was previously employed in other research studies [8, 9]. Before the analysis, the participants were divided into 3 blocks based on the chronological order. The learning curve was then assessed as an improvement of agreement between trainees and experts over time across these blocks.

### 2.1. Participants

Consecutive patients with suspected pelvic endometriosis planned for surgical treatment were enrolled in the study in a tertiary endometriosis centre. Endometriosis was suspected based on the symptoms, previous basic imaging, or findings from diagnostic laparoscopy performed in a district hospital. The inclusion criteria consisted of age 18-50 years, planned surgical treatment of pelvic endometriosis, no changes in the hormonal treatment in the last 4 months, and ultrasound and MRI to surgery time < 4 months. The exclusion criteria were age outside the desired range, suspected malignancy, delay between index imaging and surgery (reference) longer than 4 months, missing one of the 3 imaging investigations which were offered as part of the study, and/or participants declining surgery. The participants were divided into three blocks based on the order in which they were recruited. All participants underwent two ultrasound assessments, one by the ultrasound trainee and one by the ultrasound expert. Concurrently, the MRI examination was scheduled and evaluated by a radiology trainee and an expert. All four examiners were blinded to previous clinical and surgical findings and other imaging. The findings by trainees were not considered when planning for surgery.

### 2.2. Subjects

Both trainees were residents in the final years of their training, and despite having intermediate skills and experience in gynecological imaging, neither had prior experience in the assessment of endometriosis mapping (i.e., description of locations, size and numbers of DE lesions, endometriomas, adenomyosis, and frozen pelvis). The ultrasound trainee (T.I.) was a 4th year resident in O&G with intermediate ultrasound skills (3-year experience, consisting of approximately 500 gynecologic ultrasound cases), doing her postgraduate studies in endometriosis ultrasound. The radiology trainee (P.H.) was a 5th year resident in general radiology with no special interest in gynecology. The ultrasound experts (D.F., F.F.) and a radiology expert (A.B.) were all specialists in their respective fields with more than 10-year postresidency experience in advanced pelvic imaging. We did not recruit more than one sonographer trainee due to the ethical issue of subjecting participants to multiple unnecessary vaginal scans.

### 2.3. Index Tests

Both imaging modalities were reported using the ultrasound-specific protocol based on the IDEA [2] consensus. For the MRI, the protocol was adapted using some modifications [7], including removing site-specific tenderness as a soft marker and replacing sliding sign by sign of adhesions from distorted anatomy (e.g., “ear sign”). The settings and technical protocols reflected routine clinical practice. Plain transvaginal and transabdominal ultrasound examinations were performed without any bowel preparation or gel sonography using Voluson E10 (GE Medical Systems, Zipf, Austria) at a gynecology setting. The MRI assessment was done using 3 Tesla MRI Siemens scanner with a phased-array coil (Skyra, Siemens AG, Erlangen, Germany) according to the protocol recommended by the European Society of Urogenital Radiology (ESUR) [3], including the intravenous application of a spasmolytic agent, with no vaginal or rectal contrast agents.

### 2.4. Reference Standard

Trainees were assessed against two reference standards. The first standard was represented by reports from expert imaging where the trainee's diagnostic performance in the three blocks was assessed against the expert's findings. The second reference standard was a laparoscopic evaluation with histological confirmation in most cases. Anatomical sites with a normal appearance on laparoscopy were not biopsied; hence, histological confirmation was missing for those sites. Only sites judged as affected were either resected or biopsied, providing histological confirmation. Adenomyosis was not assessed on laparoscopy because only 1 patient had a hysterectomy.

### 2.5. Learning Procedure

The ultrasound trainee was assessed by ultrasound experts, and the radiology trainee was assessed by the radiology expert as being at a comparable level of their respective training. Both trainees conducted self-study prior to the study focusing on relevant guidelines and imaging protocols (IDEA [2], ESUR [3]) and a pattern recognition in endometriosis. The ultrasound trainee (T.I.) scanned patients with their consent under the indirect supervision of the ultrasound experts (F.F., D.F.) and was blinded to the clinical findings and other imaging reports. Apart from regular meetings with the supervisors and discussing cases (indirect supervision), the O&G trainee was also involved in the patients' clinical care, including assistance during surgical treatment of endometriosis providing retrospective correlation between the ultrasound and intraoperative findings. The radiology trainee (P.H.) was also blinded to the previous findings and reported MRI independently of the radiology expert (A.B.). He had regular meetings to review the imaging reports and images with the supervisor and went through operative notes retrospectively on the computer.

The learning curve was assessed as “positive” when the agreement was increasing with the increasing number of cases between the blocks and as “indeterminate” when the performance plateaued or the improvement was inconsistent.

### 2.6. Statistical Analysis

Kappa value (*k*) was used to evaluate the level of agreement between the trainees and laparoscopy/histology reference and the trainees and experts in all three blocks individually and then overall in the whole cohort. When certain anatomical sites of endometriosis involvement were missing in the block, the learning curve was calculated from 2 blocks only.

The strength of agreement was defined as follows [10]: slight (*k* < 0.20), fair (*k* 0.20–0.4), moderate (*k* 0.41–0.6), substantial (*k* 0.61–0.8), and excellent (*k* 0.81–1.0). The statistical analysis was done using SPSS with *p* < 0.05 considered statistically significant.

### 2.7. Ethical Approval

The local ethics committee approved the study protocol, and informed consent was obtained from all subjects (study number 1249/16 S-IV, approved version 1486/16 IS).

## 3. Results

### 3.1. Participants

**Figure 1:**
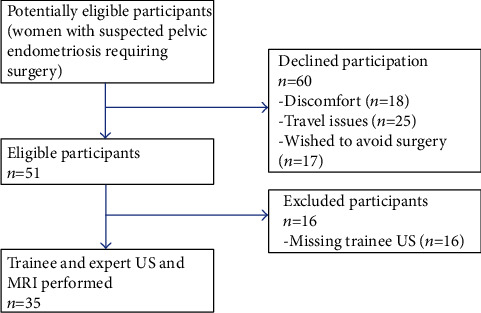
Participants flowchart. MRI: magnetic resonance imaging; *n*: number of participants; US: ultrasound.

From September 2016 to February 2018, one hundred and eleven patients were approached, but only 35 patients agreed to participate (Figure 1). The three blocks therefore contained 12 (block 1), 12 (block 2), and 11 (block 3) patients. All participants had pelvic endometriosis, although its prevalence in each of the anatomical sites varied (Table 1).

### 3.2. Learning Curves

**Figure 2:**
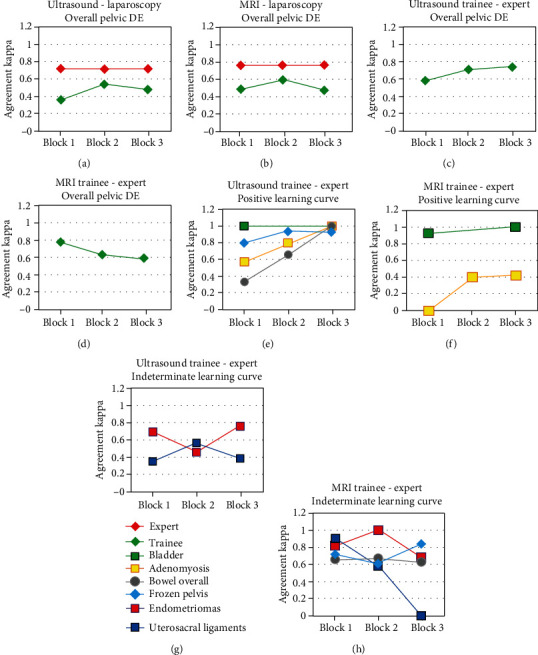
Schematic demonstration of learning curves. (a, b) Expert and trainee versus laparoscopy/histology agreement in the overall pelvic DE assessment. (c, d) Trainee versus expert agreement in the overall pelvic DE assessment. (e, f) Positive learning curves of ultrasound and radiology trainee versus expert. (g, h) Indeterminate learning curves of ultrasound and radiology trainee versus expert. DE: deep endometriosis; MRI: magnetic resonance imaging.

The results are in Tables 2 and 3 and Figure 2. The ultrasound trainee achieved a positive learning curve reaching an excellent agreement in the 3rd block in the assessment of frozen pelvis (Kappa = 0.90, *p* = 0.01), adenomyosis (Kappa = 1.00, *p* = 0.09), overall bowel assessment (Kappa = 1.00, *p* = 0.01), and bladder (Kappa = 1.00, *p* = 0.01). In the assessment of endometriomas and uterosacral ligaments, the ultrasound trainee's learning curve was indeterminate. The learning curve of the overall pelvic DE detection showed an overall improving trend, reaching substantial agreement (Kappa = 0.74, *p* = 0.01) at the end of the 3rd block.

The radiology trainee versus expert showed a statistically significant positive learning curve in adenomyosis (Kappa = 0.42, *p* = 0.09) and a bladder DE detection (Kappa = 1.00, *p* = 0.01). The radiology trainee had an indeterminate learning curve in the assessment of bowel lesions, endometriomas, uterosacral ligaments, and frozen pelvis. The learning curve of the pelvic DE detection did not show any obvious improvement and was also assessed as indeterminate.

The agreement of both trainees with expert imaging was better than the agreement with the laparoscopy in the majority of cases. Both trainees reached an excellent agreement with laparoscopy only in bladder DE detection. Both trainees failed to identify any of the 10 vaginal lesions.

## 4. Discussion

This study is the first to assess the learning curve of endometriosis assessment by ultrasound and MRI in one cohort of patients using the IDEA consensus [2]. It counts among the few studies describing the real-life learning curve for ultrasound without using offline assessments of images and/or video clips. We showed that after as few as 35 cases, the ultrasound trainee had a positive learning curve in more anatomical locations than the radiology trainee, reaching an excellent agreement in the frozen pelvis, adenomyosis, bowel, and bladder DE assessment while the radiology trainee achieved an excellent agreement in the bladder DE detection only.

Choosing ultrasound/O&G trainee and a radiology trainee reflects the typical representation of the two specialities actively involved in endometriosis imaging. Endometriosis centres can choose which imaging modality to use, but provided our results, the choice should not be solely based on the need for training in ultrasound. We show that accurate MRI reading is also dependent on the caseload, defined by its learning curve. Another strength of our study is the comparison drawn against two reference standards. In the early learning curve, it is more meaningful to compare the performance against expert imaging because it reflects the gold standard in imaging. Difficulties in detecting certain lesions (small vaginal nodules, multiple bowel lesions, etc.) will affect the accuracy of an expert, providing a performance adjustment for the trainee's accuracy. In the later learning curve, when expert levels in imaging are being reached, the comparison with laparoscopy/histology is more accurate, because ultimately, visual and histological confirmation is the gold standard in the diagnosis of endometriosis. This was demonstrated in our early learning curve, where agreement with expert imaging was achieved easier and quicker than agreement with laparoscopy.

The main limitation of our study is a small sample size where the incidence of lesions in certain anatomical sites was too low to assess a meaningful learning curve (for instance involvement of rectovaginal septum). Also, the number of trainees (2) introduces a possible bias due to personal factors. The individual learning potential of a single trainee may not be representative of a learning potential of all trainees, and any generalisation to sonographers and radiologists in training should be done with caution. In regard to data analysis, it could be argued that the use of cumulative summation tests for the learning curve (LC CUSUM) [11] might have been more appropriate. LC CUSUM offers a learning curve with a predefined threshold at which the trainee is deemed competent. In view of the limited number of cases and a small likelihood of reaching competency in all areas, we aimed to provide more graphic analysis of the development of a positive/indeterminate early learning curve, which Kappa agreement describes better. This should however have no effect on possible future comparison because even though the results are reported in different formats, they all answer the same question, which is how many cases are required to reach an expert level.

One of the interesting aspects of our study was the unexpected discrepancy in the learning curve in the ultrasound and MRI. A possible explanation for this finding lies either in individual trainees, their training, or the imaging modality itself. The first is related to the individual learning ability of the trainees, their speed of internalizing new information, and skillset. From the training perspective, although both trainees received feedback on their reporting skills, the O&G trainee was directly involved in providing medical care to the participants. We assume that the learning of the ultrasound trainee was enhanced by their involvement in other aspects of the endometriosis care, such as direct contact with the patients, multidisciplinary meetings, and assistance in theatres with a possibility to correlate the real-life appearance of pelvic endometriosis with ultrasound images. The third aspect is a possible enhanced learning in ultrasound as an imaging modality, stemming from the combination of soft markers (such as site-specific tenderness) and the imaging method itself. Tenderness during ultrasound examination guides the sonographer to the points of likely involvement, increasing the chances of detecting small nodules, such as on uterosacral ligaments and bowels. Although it was not in the design of this study, we can presume that adding clinical examination (such as bimanual palpation) to the ultrasound examination would enhance the training as well, making the sonographer/gynecologist's learning curve even steeper.

Another unexpected finding was the inconsistent trainees' accuracy in detecting endometriomas, worse agreement with experts than with laparoscopy. On retrospective review of all the cases, experts reported in more details, including small endometriomas, which were ignored at the surgery, while the trainees tended to focus on bigger lesions which in turn explained the seemingly better agreement trainee-laparoscopy in the endometrioma assessment. There were also two cases of ovarian abscess, where intracystic content of ground glass appearance is not distinguishable from endometrioma on the ultrasound but is easy to differentiate on the T1 and T2 MRI sequences. This resulted in a better diagnostic performance of the MRI trainee in the first block.

Previous research assessed the learning curve in endometriosis mapping in several ways. Guerriero et al. [11] assessed the learning curve on offline and hands-on training and suggested that between 17 cases (bladder DE) and 44 cases (uterosacral ligaments) are required to reach a predefined threshold in accuracy. Extrapolated to our study, it represents approximately 100-150 cases to achieve a plateau in all areas, and our 35 cases therefore truly correspond to the early stages of the learning curve of DE assessment. Bazot et al. [12] however showed on the learning curve of ultrasound assessment of endometriomas that the inter-trainee variability was very wide and suggested that the assessment of the learning curve might require a more individual approach in training, rather than standardise a set number of cases for everyone in training.

Future studies should address the learning curve in pelvic endometriosis assessment in its entirety with a hands-on setting, preferably undertaken in tertiary centers to ensure a steady flow of disease-positive cases. Since the learning curve is not a uniform entity for all trainees, employing several trainees in one study would be beneficial to define a range of cases required to achieve competency. This should then be reflected in the requirements for endometriosis centre accreditation.

In conclusion, this unique study comparing the early learning curve of an O&G trainee using ultrasound and a radiology trainee using MRI when evaluating pelvic endometriosis showed a positive learning curve in several areas in as little as 35 cases. A bigger caseload would be required to demonstrate the learning curve in full. Secondly, we found that the ultrasound trainee had positive learning curves in more anatomical locations (bladder, adenomyosis, overall bowel DE, frozen pelvis) than the radiology trainee (bladder, adenomyosis), which could be down to individual factors, the difference in training, or the imaging method itself.

## Figures and Tables

**Table 1:** Prevalence of the affected anatomical sites per block (subcohorts of participants based on the chronology in the recruitment).

Endometriosis location	Number of cases (total)	Block 1	Block 2	Block 3
Frozen pelvis	29	9	11	9
Uterosacral ligaments	25	6	8	11
Bowel (rectum, rectosigmoid)	19	5	7	7
Endometriomas	18	8	2	8
Vagina	10	2	3	5
Adenomyosis	9	2	4	3
Bladder	8	6	2	0
Rectovaginal septum	4	2	1	1

**Table 2:** Agreement with experts and laparoscopy.

		Trainee–expert agreement						Trainee–laparoscopy/histology agreement					
		Ultrasound			MRI			Ultrasound			MRI		
Patients in 3 blocks (total number of lesions)		1st block	2nd block	3rd block	1st block	2nd block	3rd block	1st block	2nd block	3rd block	1st block	2nd block	3rd block
Frozen pelvis overall	29	0.800	0.941	0.933	0.721	0.609	0.836	0.351	0.713	0.800	0.044	0.027	0.267
Uterosacral ligaments	25	0.357	0.571	0.394	0.903	0.583	-0.023 (NS)	0.231	0.100	0.191 (NS)	0.474	0.489	0.083 (NS)
Bowel (R, RS)	19	0.330 (NS)	0.657	1.000	0.657	0.667 (NS)	0.621	0.333 (NS)	0.657	0.560	0.471 (NS)	0.833 (NS)	0.298 (NS)
Endometriomas	18	0.697	0.467	0.76	0.817	1.000	0.681	0.697	0.647	0.783	0.814	0.625	0.681
Adenomyosis	9	0.571 (NS)	0.800 (NS)	1.000 (NS)	0 (NS)	0.400 (NS)	0.421 (NS)	Not computerised^∗^					
Vagina	10	Not computerised^∗^											
Rectovaginal septum	4	Not computerised^∗^											
Total pelvis		0.583	0.708	0.735	0.784	0.627	0.592	0.397	0.542	0.483	0.479	0.592	0.474
Patients in 2 blocks		1st block	2nd block		1st block	2nd block		1st block	2nd block		1st block	2nd block	
Bladder	8	1.000	1.000		0.824	1.000		0.667	1.000		0.667	1.000	

DE: deep endometriosis; MRI: magnetic resonance imaging; R: rectum; RS: rectosigmoid. NS: statistically not significant result (*p* > 0.05). ^∗^Adenomyosis was not assessed against surgical reference standard because only 1 patient had a hysterectomy, vaginal DE not was computerised since none of the 10 lesions were detected on the trainee imaging, and rectovaginal septum DE not was computerised due to low prevalence (2 nodules in the 1st block, 1 lesion in the 2nd block, 1 lesion in the 3rd block).

**Table 3:** Overall performance in the learning curve.

r	Interobserver agreement			
	Trainee/expert (1st reference)		Trainee–laparoscopy (2nd reference)	
	Ultrasound	MRI	Ultrasound	MRI
Frozen pelvis	0.903 (*p* 0.00)	0.735 (*p* 0.00)	0.623 (*p* 0.00)	0.128 (*p* 0.00)
Uterosacral ligaments	0.512 (*p* 0.00)	0.601 (*p* 0.00)	0.261 (*p* 0.01)	0.455 (*p* 0.00)
Bowel (rectum, rectosigmoid)	0.633 (*p* 0.00)	0.699 (*p* 0.00)	0.539 (*p* 0.00)	0.598 (*p* 0.00)
Endometriomas	0.706 (*p* 0.00)	0.828 (*p* 0.00)	0.754 (*p* 0.00)	0.746 (*p* 0.00)
Vagina	Not computerised^∗^			
Adenomyosis	0.769 (*p* 0.00)	0.279 (*p* 0.00)	Not computerised^∗^	
Bladder	1.0 (*p* 0.00)	0.717 (*p* 0.00)	0.800 (*p* 0.00)	0.717 (*p* 0.00)
Rectovaginal septum	Not computerised^∗^			
Pelvic DE overall	0.690 (*p* 0.00)	0.697 (*p* 0.00)	0.490 (*p* 0.00)	0.531 (*p* 0.00)

Agreement between trainees and experts and trainees and laparoscopy/histology in the overall assessment of endometriosis in all 3 blocks, expressed in Kappa value. DE: deep endometriosis; p: *p* value; POD: pouch of Douglas; R: rectum; RS: rectosigmoid; USL: uterosacral ligament. ^∗^None of the 10 vaginal lesions were detected correctly by the trainees; only 4 rectovaginal septum lesions in the cohort out of which none was identified by the ultrasound trainee and only one correctly identified by the radiology trainee; adenomyosis detection was not assessed against laparoscopy/histology since only 1 patient had a hysterectomy.

## Data Availability

The data that support the findings of this study are available from the corresponding author, AB, upon reasonable request.
